# Lifestyle, Physical Activity, Eating and Hygiene Habits: A Comparative Analysis Before and During the COVID-19 Pandemic in Student Population

**DOI:** 10.3389/fpubh.2022.862816

**Published:** 2022-03-17

**Authors:** Marija Sekulic, Dalibor Stajic, Aleksandra Jurisic Skevin, Aleksandar Kocovic, Radica Zivkovic Zaric, Nela Djonovic, Dragan Vasiljevic, Branimir Radmanovic, Marko Spasic, Katarina Janicijevic, Ivana Simic Vukomanovic, Jovan Niciforovic, Katarina Parezanovic Ilic, Stevan Barac, Tanja Lukovic, Stefan Joksimovic

**Affiliations:** ^1^Department of Hygiene and Ecology, Faculty of Medical Sciences, University of Kragujevac, Kragujevac, Serbia; ^2^Department of Physical Medicine and Rehabilitation, Faculty of Medical Sciences, University of Kragujevac, Kragujevac, Serbia; ^3^Department of Pharmacy, Faculty of Medical Sciences, University of Kragujevac, Kragujevac, Serbia; ^4^Department of Pharmacology and Toxicology, Faculty of Medical Sciences, University of Kragujevac, Kragujevac, Serbia; ^5^Department of Psychiatry, Faculty of Medical Sciences, University of Kragujevac, Kragujevac, Serbia; ^6^Department of Surgery, Faculty of Medical Sciences, University of Kragujevac, Kragujevac, Serbia; ^7^Department of Social Medicine, Faculty of Medical Sciences, University of Kragujevac, Kragujevac, Serbia; ^8^Department of Natural Sciences, Faculty of Hotel Management and Tourism in Vrnjacka Banja, University of Kragujevac, Vrnjacka Banja, Serbia; ^9^Department of Forensic Psychiatry, University Clinical Centre Kragujevac, Kragujevac, Serbia; ^10^Surgical Oncology Clinic, Institute for Oncology and Radiology of Serbia, Belgrade, Serbia

**Keywords:** coronavirus, lockdown, sedentary behavior, nutrition, personal hygiene habits

## Abstract

**Background:**

Changing daily habits such as diet, hygiene and physical activity may be some of the consequences of the COVID-19 pandemic. The aim of the study was to analyze the effect of this pandemic on lifestyle, physical activity, eating and hygiene habits among students.

**Methods:**

This cross-sectional study involved 171 students from the Faculty of Medical Sciences, University of Kragujevac, Serbia. Data were statistically analyzed using Wilcoxon Signed-Rank test, Marginal homogeneity test and Chi-square test. The differences were considered statistically significant when *p* ≤ 0.05.

**Results:**

In this study, it was observed that the most common physical activity before the pandemic was walking, while during the pandemic was home exercising. Compared to the period before the pandemic, there was no difference in the time spent engaging in daily physical activity (*p* = 0.334). However, there was a significant increase in sitting time during the pandemic (*p* = 0.005). Difference was noticed in the use of breakfast, the number of meals, and the type of fat in the diet before and during the pandemic (*p* = 0.000). During the pandemic, there was an increase in the use of fruits (*p* = 0.000), vegetables, and nuts (*p* = 0.001), while the use of fast food and alcohol have decreased. During the COVID-19 pandemic, a significant increase in the use of dietary supplements was observed (40.2%), (*p* = 0.008).

**Conclusions:**

Given that the COVID-19 pandemic is ongoing, certain changes in lifestyle observed in this study should be confirmed in more extensive population studies.

## Introduction

The Coronavirus Disease 2019 (COVID-19) is a severe acute respiratory syndrome caused by SARS coronavirus 2 (SARS-CoV-2). The first series of pneumonia cases were described in Wuhan, Hubei Province, China in December 2019. COVID-19 has rapidly spread to other parts of the world causing the pandemic (1). With the growing case notification rates at Chinese and other international locations, and Serbia was also affected, with the first confirmed case on March 6, 2020 (2, 3). The government of Serbia declared a state of emergency on March 15, 2020, implementing some of Europe's strictest measures to combat the pandemic (such as 12 h and weekend police-enforced curfew, strict bans on movement, and shutting borders) (4).

Due to the introduction of various measures to bring the pandemic under control, sudden and radical changes in the habits and lifestyle of the population occurred, with a significant reduction in any form of socialization. Educational institutions such as kindergartens, schools and universities were completely suspended, and students were staying indoors for months and learning via TV and online platforms (4). Self-isolation and physical distance have affected the lives of citizens, especially their daily behavior and eating habits. Although restrictions such as social distancing, reduced group gatherings, closed gyms, etc. are efficient in reducing the infection rate, such restrictions result in limiting participation in normal daily activities, travel, and physical activity. The prolonged homestay may lead to increased sedentary behaviors, such as spending excessive amounts of time sitting or lying down that consequently makes it difficult to fight infection, and lead to immunological and cardiopulmonary complications of more severe outcomes (5).

Optimal level of nutrition, food choices as well as eating habits have long-term benefits on disease prevention, including COVID-19, and may affect the course and outcome of COVID-19 infection. Studies show that consuming healthy foods has a rapid anti-inflammatory effect, even in the presence of obesity pathology (6).

Given the ease and speed of human-to-human transmission of the virus, Non-pharmaceutical measures such as wearing face masks and washing and disinfection hands play an important role in the risk of transmission because they establish a barrier to aerosol spread and protect vulnerable populations (7, 8). Evidence from the literature showed that frequent hand-washing would reduce the risk of viral transmission by 55% (7).

Although the COVID-19 pandemic and the lockdown have induced changes in all aspects of life and the living habits of people of all ages, young people were particularly affected by these changes. However, not enough research has been conducted to investigate the effect of the pandemic on student population in Serbia. The aim of this study was to analyze the differences in lifestyles, eating, and hygiene habits before and during the pandemic among the students (aged ≥ 19) of the Faculty of Medical Sciences in Kragujevac, Serbia.

## Method

This study is designed as a cross-sectional study in the period from June 1st until August 1st, 2020. It included 171 students from the Faculty of Medical Sciences, University of Kragujevac, Serbia (128 female respondents and 43 male respondents, aged ≥ 19). The study comprised a structured questionnaire which was available on the online platform of the faculty, disseminated through institutional networks, accessible through any device with an Internet connection from June 1st until August 1st, 2020. The questionnaire was also provided in paper form for students who came to the faculty during this period to take exams, who did not complete the online questionnaire. Students agreed to participate in an anonymous survey. The study was approved by the institution where the research was conducted. The questions were divided into different sections: socio-demographic data (age, gender); anthropometric data (weight and height, waist circumference); information on eating habits (regularity of breakfast, number of daily meals, type of bread used, frequency of use of dairy products, type of fat used for food preparation, type of meat, salting, type and frequency of fruit and vegetable consumption, method of food preparation); information on life habits (alcohol use; physical activity–form and time period of physical activity) and hygiene habits. The questionnaire was in online format and paper form. Body mass index value (BMI) was used to assess nutritional status and divide respondents into weight status categories–underweight, normal weight, overweight, and obesity. After completing the questionnaire on the Google platform, the data were downloaded to the Microsoft Excel sheet. These data are combined with the data from the questionnaire in the paper version.

Statistical analyzes were performed using the SPSS program, version 20.0. The analysis of the normality of the distribution of continuous variables was performed using the Kolmogorov-Smirnov test. The significance of differences between continuous variables before and during the COVID-19 pandemic was determined using the Wilcoxon Signed-Rank test, as the variables did not follow the normal distribution. The Marginal homogeneity test was used to determine the difference between these two periods for categorical variables. The Chi-square test was used to determine the difference between the sexes in certain variables. Statistical significance existed when *p* ≤ 0.05.

## Results

The average age of the respondents was 22.53 (*SD* = 2.477), and the average height of the respondents was 161.86 cm (*SD* = 36.807). During the pandemic, there was no significant change in average values for body weight (Wilcoxon Signed Rank test *p* = 0.755) and waist circumference (Wilcoxon Signed Rank test *p* = 0.979) compared to the period before the pandemic (Table 1). A significant percentage of male subjects were overweight both before (Pearson chi-square value = 10.518, *df* = 2, *p* = 0.005) and during the pandemic (Pearson chi-square value = 14.106, *df* = 3, *p* = 0.003) compared to female subjects. No significant difference in BMI (*p* = 0.568, Wilcoxon Signed Rank test) was found before and during the pandemic (Tables 1, 2).

**Table 1:** Data on age, height, weight, BMI, and waist circumference of respondents, differences before and during the COVID-19 pandemic (Wilcoxon Signed Rank test).

	***N***	**Minimum**	**Maximum**	**Mean**	**Std. deviation**	***p*-value**
Age	171	19	37	22.53	2.48	
Grade Point Average	167	7	10.00	8.44	0.79	
Weight before pandemic (kg)	171	42	108	65.04	12.63	0.755
Weight during the pandemic (kg)	172	43	110	65.02	12.90	
Height (cm)	153	2	193	163.69	36.50	
BMI before the pandemic (kg/m^2^)	144	16.33	29.75	21.77	2.91	0.568
BMI during the pandemic (kg/m^2^)	145	16.16	30.85	21.69	2.87	
Waist circumference before the pandemic (cm)	120	50	125	73.85	13.89	0.979
Waist circumference during the pandemic (cm)	119	50	128	73.45	14.16	

**Table 2:** Nutritional status (BMI), χ^2^ test by gender before COVID-19 (Pearson chi-square value = 10.518, *df* = 2, *p* = 0.005) and during COVID-19 pandemic (Pearson chi-square value = 14.106, *df* = 3, *p* = 0.003).

**COVID-19 pandemic**	**Before**	**During**	**Before**	**During**	**Before**	**During**	**Before**	**During**
**BMI (m/kg^**2**^)**	**Underweight %**		**Normal weight %**		**Pre-obesity %**		**Obesity %**	
Total	11.8	9	74.0.3	77.8	13.9	11.8	0	1.4
Males	2.9	2.9	68.6	68.6	28.6	28.6	0	0
Females	14.7	11	76.1	80.7	9.2	6.4	0	1.8

When it comes to eating habits, there was no difference in the use of bread before and during the pandemic (Marginal homogeneity test *p* = 0.881), there was a significant difference in the consumption of breakfast, and the number of meals, type of fat used by respondents before and during the pandemic (*p* = 0.000) (Table 3). Before the pandemic, 64% of respondents had breakfast every day, and during the pandemic 84.3% of respondents. The percentage of respondents who had 5 or more meals during the day increased from 15.6 to 28.9% of respondents. The use of lard increased from 16.4 to 27.5%. There was no difference in the consumption of pork (*p* = 0.752), beef (*p* = 0.515), poultry (*p* = 0.285), fish (*p* = 0.782) and cured meat products (*p* = 0.913) before and during the pandemic.

**Table 3:** Overview of the percentage of respondents' responses regarding eating habits and supplementation in which a significant difference was found before and during the COVID-19 pandemic (Marginal homogeneity test).

**% of respondents with a certain answer**			
**COVID-19 pandemic**	**Before the COVID-19 pandemic (%)**	**During the COVID-19 pandemic (%)**	**Marginal homogeneity test** ***p*****-value**
1. Breakfast			*p* = 0.000
Every day	64	84.3	
Sometimes	30.8	13.4	
Never	5.2	2.3	
2. Number of meals during the day			*p* = 0.000
<3	28.9	15.6	
3	34.7	31.8	
4	20.8	23.7	
5	11.6	23.1	
>5	4	5.8	
3. The type of fat used in the diet			*p* = 0.000
Lard	16.4	27.5	
Butter	1.8	1.2	
Vegetable fat	7.6	6.4	
Margarine	3.5	2.9	
Oil	68.4	59.6	
None	2.3	2.3	
4. Consumption of fruit			*p* = 0.000
Once or more a day	25	33.7	
4–6 times a week	23.3	28.5	
1–3 times a week	37.2	29.1	
<1 per week	13.4	7.6	
Never	1.2	1.2	
5. Consumption of vegetables			*p* = 0.001
Once or more a day	40.6	45	
4–6 times a week	32.9	36.7	
1–3 times a week	24.1	16.6	
<1 per week	2.4	1.8	
Never	0	0	
6. Consumption of nuts			*p* = 0.001
Yes	37.1	44.1	
Not	12.4	15.3	
Sometimes	50.6	40.6	
7. Method of food processing			
Baking	50.6	64.3	*p* = 0.000
Cooking	72.6	82.7	*p* = 0.002
Frying	66.1	72	*p* = 0.041
Stew	27.4	33.3	*p* = 0.025
Raw food	29.2	35.7	*p* = 0.016
Fast food	35.7	17.9	*p* = 0.000
8. Alcohol use			*p* = 0.022
Once or more times a day	1.2	1.2	
4–6 times a week	1.2	2.5	
1–3 times a week	11.7	9.8	
<1 per week	42.9	33.7	
Never	42.9	52.8	
9. Consumption of dietary supplements			*p* = 0.008
Yes	33.7	40.2	
Not	66.3	59.8	

There was a significant increase in the consumption of fruits (*p* = 0.000), vegetables, and nuts (*p* = 0.001) during the pandemic. Daily consumption of fruits increased from 25 to 33.7%, vegetables from 40.6 to 45%, and nuts from 37.1 to 44.1% of respondents (Table 3). No difference was observed in the consumption of certain types of fruit, except in the case of the use of lemon (*p* = 0.000), which was previously used by 39.6% of respondents, and during the pandemic by 50.6%.

The method of food preparation before and during the pandemic differed significantly. There was a significant difference in food processing by baking (*p* = 0.000), cooking (*p* = 0.002), frying (*p* = 0.041), stewing (*p* = 0.025), use of raw food (*p* = 0.016) and use of fast food (*p* = 0.000). The percentage of all types of food processing increased, with cooking being the most common, food frying increased from 66.1 to 72%, while the consumption of fast food decreased from 35.7 to 17.9%. There was no difference in the consumption of coffee (*p* = 0.891) and carbonated beverages (*p* = 1.00), while the consumption of alcohol decreased significantly (*p* = 0.022).

During the COVID-19 pandemic, a significant increase in the use of dietary supplements, which was not prescribed by a physician, was observed (40.2%), compared to the period before the pandemic (33.7%, *p* = 0.008) (Table 3). In particular, an increase in the use of supplements containing vitamins C (*p* = 0.028), D (*p* = 0.002) and zinc (*p* = 0.000) was observed. Before the pandemic, 66% used vitamin C, 12.8% vitamin D, and 30.8% zinc, while during the pandemic the use of vitamin C was recorded in 73.3%, vitamin D in 21.1%, and zinc in 47.2% of respondents. However, there was no difference in the use of B vitamins (*p* = 0.239) and calcium (*p* = 0.096).

During the pandemic, more frequent thinking about health was observed when choosing food and planning a diet (*p* = 0.000).

Compared to the period before the pandemic, there was no significant difference in the time spent engaging in physical activity during the day (*p* = 0.334). The most common activity before the pandemic was walking, and during the pandemic, home exercising. Walking decreased from 40.7 to 24.2% of respondents during the pandemic. There was a significant increase in sitting time (average 5.73 h) compared to the Pre-pandemic period (average 5.29 h), (Wilcoxon test, *p* = 0.005).

When it comes to hygienic habits that help protect against coronavirus infection, it was found that during the pandemic, 90.9% washed their hands before meals, 93.9% after using the toilet, 89% after coming home, and 38.4% of respondents before putting on masks. The largest percentage of respondents used soap and hot water for handwashing (97%), while 2.4% of respondents used alcohol-based products for dry hand washing. About 30% of respondents touched their nose, mouth, and eyes outside the house with unwashed hands. In public, 31.1% of respondents always wore a mask, while 53.4% respondents wore a mask often, 13% of them wore it rarely, and 2.5% of respondents never wore it. The largest percentage of respondents (81%) used medical-surgical masks, 39.9% of respondents wore masks made of cotton, linen or other materials, while epidemiological masks N95 were used by 9.2% of respondents. The highest percentage of respondents (40.7%) used the same mask (single or multiple without washing and disinfection) only 1 day, 32.1% of respondents used the mask for up to 3 days, more than 5 days used the same mask 9.3% of respondents, while 2–4 h the same mask was used by 7.4% of respondents. There were 76.1% of respondents who used alcohol to disinfect their hands when they were away from home, 57.7% after shopping, 46% after using an ATM, and 27.6% after visiting a pharmacy. 33.7% of respondents have never used surgical gloves outside the home when handling money and shopping.

During the pandemic, a significantly higher percentage (50.3%) of respondents cleaned and disinfected their shoes after returning home (*p* = 0.001) compared to the period before the pandemic (11%). During the pandemic, a significant percentage (50%) of respondents took showers and changed clothes upon returning home, compared to the Pre-pandemic period (27.8%) (*p* = 0.000).

Compared to the period before the pandemic, there was a significant difference in cleaning, washing, and disinfection of food after purchase (*p* = 0.000), before the pandemic 6.1% of respondents treated food in this way, and during the pandemic 35.6% of respondents.

The recommendation to maintain the required social distance was always followed by 30.7% of respondents, often 55.2%, rarely 12.9%, and never 1.2% of respondents.

Respondents obtained the information on the COVID-19 pandemic through the Internet (89.2%) and television (78.4%).

During the pandemic, a significant decrease in household income was observed compared to the period before the pandemic (*p* = 0.049, Wilcoxon Signed Rank Test).

## Discussion

The COVID-19 pandemic encouraged researchers to examine what eating habits, physical activity, and hygiene habits were before and whether there were changes during the pandemic, taking into account the socioeconomic and sociodemographic characteristics of the respondents.

The study observed a significant decrease in household income during the pandemic compared to the Pre-pandemic period, which is probably due to job loss or lower-income during the isolation period. Although the COVID-19 pandemic affects populations around the world, there are numerous reports documenting a greater impact of the pandemic on lower socioeconomic groups (9). The risk of unemployment is higher for those with atypical and precarious employment conditions, whose financial income is already minimal. While unemployment as a whole is rising, low-income people are more likely to work in the sectors hardest hit by the pandemic and have fewer economic reserves to maintain a period of lost income, and the negative impact of unemployment is reflected in health. The prevalence of the disease is inversely related to socioeconomic status (10).

Nutritional status can have a significant impact on an individual's general health (11, 12). These changes in dietary patterns during quarantine can potentially lead to changes in body weight as a result of lower physical activity, changes in food consumption, and stress, which may be due to adaptation to the new situation (13). Following the bodyweight of the student population, there is no significant deviation in the average values of body weight, but it is noted that a significant percentage of male respondents were overweight before and during the pandemic compared to female respondents, which is consistent with previous studies which examined gender differences in the prevalence of overweight and obesity among university students (14, 15).

When it comes to eating habits, during the COVID-19 pandemic, there is a noticeable increase in respondents who ate breakfast every day and had more meals during the day. Similar findings are evident in a study conducted in Poland, where the majority of respondents, 65.5%, stated that they practice breakfast every day. Numerous studies document that skipping breakfast can be associated with an increased risk of various diseases, and given that this information is available in various sources for health promotion that are more commonly reached by individuals with higher education, it is not surprising the frequency of breakfast (13).

There was also a significant difference in food preparation before and during the pandemic, with an increase in the percentage of all food processing, where cooking was the most common form of preparation, while the consumption of fast food decreased dramatically from 35.7 to 17.9%. It is assumed that one of the reasons for choosing this method of food preparation is greater information of the population about the importance of healthy food preparation, as a preventive measure to prevent various diseases. During the pandemic, the study showed a significant increase in consumption of fruits, vegetables, and nuts, which is documented by the findings of research conducted in the regions of Spain, Brazil, Chile (16), and Italy, where 37.4% of the population said they increased consumption of healthy food (17). Improving the quality of nutrition was also noted by Grant et al. in their extensive study of 2,678 people (18). These results are not surprising given that due to confinement, families have had more time to improve their eating habits by increasing their intake of legumes, fruits, and vegetables and the fact that the WHO recommends fruits and vegetables as the best food during self-quarantine or longer stays at home (16). However, some people have reduced their use of fruits and vegetables, citing difficulties in finding open food stores in the immediate area, Scarmozzino et al. point out in their study. When it comes to determining the difference in the use of beverages such as coffee and carbonated beverages, it is not recorded during the pandemic compared to the previous period. However, there is a significant reduction in alcohol use, which is probably a consequence of the inability to go to cafes and nightclubs where younger individuals are surrounded by other peers who consume alcohol. Similar findings were reported in a study conducted in Italy (19).

Food and micronutrients, especially vitamins C, D, and zinc, support the body in the fight against viral agents (20). Thus, in this research, during the pandemic, a significant increase in the use of dietary supplements was observed, especially those that contain vitamins C, D, and zinc, without being prescribed by a doctor, but were the choice of the respondents. Vitamin C deficiency impairs immune function and increases susceptibility to infections (21). The results of meta-analyses of 39 studies from around the world document an increased risk of SARS-CoV-2 infection and a more severe clinical outcome due to vitamin D deficiency (22). Improved immunity, support in the fight against infection, and inflammation are thought to have been the main reasons for increasing the use of these supplements (23).

Following the time spent in physical activity during the day in the period before and during the pandemic, no significant difference is observed. The most common activity before the pandemic was walking, while during the pandemic there was an exercise at home. In our research, during the pandemic, a significant decrease in walking was observed. These findings are in line with the results of the previous studies (24, 25). Despite the fact that there were restrictions on movement, the results of the research by López-Valenciano et al. indicate that students who were previously active continued to be during quarantine (25). However, the research shows an increase in sitting time compared to the period before the pandemic, which is confirmed by the research of Ammar et al., which shows an increase in daily sitting time by more than 28%, probably due to the negative impact of COVID-19 home confinement (5).

Numerous studies have been conducted with the aim of examining hygiene habits and the existence of changes in them in order to prevent further development of the COVID-19 pandemic. The student population of this study followed the recommended anti-epidemic measures. The largest percentage of respondents (81%) used medical-surgical masks while epidemiological N95 masks were used by 9.2% of respondents, with 53.4% of respondents often wearing a mask in public. Hand hygiene is one of the important interventions in the prevention of infectious diseases. In order to maintain hand hygiene, 97% of respondents used soap and warm water, while 76.1% of respondents used alcohol to disinfect their hands when they were away from home. It is assumed that public awareness of the importance of hygiene habits during this pandemic will have an important impact on global hygiene habits and that the habit of using hand sanitizers will remain after the COVID-19 pandemic as an integral part of proper personal hygiene (26).

Given that the COVID-19 pandemic is ongoing, certain changes in lifestyle observed in this study should be confirmed in more extensive population studies.

The main limitation of this study is the self-reported questionnaire, which can lead to actual misreporting of data related to independent assessment of body weight, height and waist circumference of the respondents. Measuring the physical activity of the respondents could also be done with more objective assessment tools, but due to the pandemic reasons, there have been limitations in more accurate and general collection of these data. The strength of our research was the fact that the research was conducted quickly after the lockdown, in the most critical period of the epidemic in Serbia.

## Data Availability Statement

The raw data supporting the conclusions of this article will be made available by the authors, without undue reservation.

## Ethics Statement

Ethical review and approval was not required for the study on human participants in accordance with the local legislation and institutional requirements. The patients/participants provided their written informed consent to participate in this study.

## Author Contributions

All authors listed have made a substantial, direct, and intellectual contribution to the work and approved it for publication.

## Conflict of Interest

The authors declare that the research was conducted in the absence of any commercial or financial relationships that could be construed as a potential conflict of interest.

## Publisher's Note

All claims expressed in this article are solely those of the authors and do not necessarily represent those of their affiliated organizations, or those of the publisher, the editors and the reviewers. Any product that may be evaluated in this article, or claim that may be made by its manufacturer, is not guaranteed or endorsed by the publisher.
